# Modeling and Learning Constraints for Creative Tool Use

**DOI:** 10.3389/frobt.2021.674292

**Published:** 2021-11-05

**Authors:** Tesca Fitzgerald, Ashok Goel, Andrea Thomaz

**Affiliations:** ^1^ Robotics Institute, Carnegie Mellon University, Pittsburgh, PA, United States; ^2^ School of Interactive Computing, Georgia Institute of Technology, Atlanta, GA, United States; ^3^ Department of Electrical and Computer Engineering, University of Texas at Austin, Austin, TX, United States

**Keywords:** tool manipulation, tool transfer, learning from corrections, human-robot interaction, cognitive robotics

## Abstract

Improvisation is a hallmark of human creativity and serves a functional purpose in completing everyday tasks with novel resources. This is particularly exhibited in tool-using tasks: When the expected tool for a task is unavailable, humans often are able to replace the expected tool with an atypical one. As robots become more commonplace in human society, we will also expect them to become more skilled at using tools in order to accommodate unexpected variations of tool-using tasks. In order for robots to creatively adapt their use of tools to task variations in a manner similar to humans, they must identify tools that fulfill a set of task constraints that are essential to completing the task successfully yet are initially unknown to the robot. In this paper, we present a high-level process for tool improvisation (tool identification, evaluation, and adaptation), highlight the importance of tooltips in considering tool-task pairings, and describe a method of learning by correction in which the robot learns the constraints from feedback from a human teacher. We demonstrate the efficacy of the learning by correction method for both within-task and across-task transfer on a physical robot.

## 1 Introduction

The abundant use of tools for a large range of tasks is a hallmark of human cognition (Vaesen, 2012). Design of new tools for accomplishing novel tasks, as well as improvisation in the absence of typical tools and use of tools in novel ways, are characteristics of human creativity. Consider for example, the design of a paperweight to hold a sheaf of papers, or the use of a paperweight to hammer in a nail if an actual hammer is not available. Both require reasoning about complex relationships that characterizes human cognition and creativity (Penn et al., 2008): The latter task, for instance, requires reasoning about the relationships among the force required to hammer in a nail, the surface of the nail’s head, the surface of the paperweight bottom, the weight of the paperweight, and so on.

A robot situated in human society will also encounter environments and tasks suited for human capabilities, and thus it is important for a robot to be able to use human tools for human tasks (Kemp et al., 2007). While a robot may learn to complete a new task with a new tool *via* demonstrations by a human teacher (Argall et al., 2009; Rozo et al., 2013), the demonstration(s) provided for that tool cannot prepare the robot for all variations of that tool it is likely to encounter. These variations can range from different tool dimensions (e.g., different sized spoons, hammers, and screwdrivers) to tool replacements when a typical tool is not available (e.g., using a measuring cup instead of a ladle, or a rock instead of a hammer). An additional challenge is that tools are often used to manipulate other objects in the robot’s environment. Given that the shape of a tool alters its effect on its environment (Sinapov and Stoytchev, 2008), a tool replacement may necessitate a change in the manipulation of that tool in order to achieve the same task goal (Brown and Sammut, 2012).

One aim of developing *creative robots* is to enable robots to exhibit creative reasoning in a similar manner as humans in order to enhance human-robot collaboration. Recently, Gubenko et al. (2021) have called for an interdisciplinary approach that synthesizes conceptual frameworks from diverse disciplines such as psychology, design, and robotics to better understand both human and robot creativity. In human cognition, creative reasoning is exemplified by improvised tool use; particularly, our ability to use analogical reasoning to identify replacement tools or methods that may be used to achieve the original goal, as well as reason over the differences between the original and replacement approaches in order to adapt the replacement to the task (Goel et al., 2020). In design, for example, there is the notion of intrinsic functions and ascribed functions (Houkes and Vermaas, 2010): In the latter, the user can use the object or tool for an ascribed function. Our goals for creative robots are similar: to be able to reason over the suitability of possible tool replacements when the original tool is unavailable, and reason over how the robot’s execution of the task must be adapted for the replacement tool.

There are several key challenges in enabling robots to creatively use new tools. First, the robot must **explore** novel tool replacements that support the task constraints. Second, the robot must be able to **evaluate** a novel tool’s suitability for a particular task, which involves learning a model of the interactions between the robot’s gripper, the tool, objects in the robot’s environment that are manipulated by that tool, and how those interactions affect the completion of the task goals. Finally, the robot must **adapt** its task model to the novel tool in order to fulfill these constraints. Prior work has addressed these first two challenges by constructing or identifying creative tool replacements (Choi et al., 2018; Sarathy and Scheutz, 2018; Nair and Chernova, 2020). In this paper, we identify and model the tooltip constraints that play a role in all three of these challenges. In particular, we focus on the third challenge of adapting a robot’s task model to a novel tool. The contributions of this paper are as follows:

1) An exploratory analysis of the manipulation constraints that must be fulfilled when using a tool to complete three tasks in simulation.
2) Two models that represent the relationship between the orientation and position constraints when manipulating a tool.
3) An algorithm for training these models using interaction corrections provided by a human teacher, first proposed in Fitzgerald et al. (2019).
4) A discussion of the generalizability of these models when applied to new tools and/or tasks.

We organize the rest of this paper as follows. Section 2 presents a summary of related work in cognitive science, computational creativity, and robotic tool use. Section 3 defines the tool transfer problem in terms of constraints on the tooltip pose, which we then explore in Section 4 *via* an extensive evaluation of the effect of tooltip perturbations on task performance in simulation. In Section 5, we discuss how a robot may learn these constraints through corrections provided *via* interaction with a human teacher. Finally, we summarize this paper in Section 6.

## 2 Background

### 2.1 Defining Creative Reasoning

What does it mean for a robot to be “creative”? Prior work in creative robotics has often fallen under one of two categories of creativity: 1) Producing a creative output involving creative domains such as music (Gopinath and Weinberg, 2016) and painting (Schubert and Mombaur, 2013), or 2) Invoking a creative reasoning process. Within the latter category, several criteria for creative reasoning have been proposed, such as autonomy and self-novelty (Bird and Stokes, 2006), in which the robot’s creative output is novel to itself but not necessarily to an outside observer. Another definition of a creative reasoning process is one that emphasizes both the variation of potential solutions considered by the agent, as well as the process used to consider and select from those options (Vigorito and Barto, 2008).

Creative reasoning may also be defined in an interactive setting. Co-creativity is a process for creative reasoning in which an agent interacts with a human to iteratively improve upon a shared creative concept. In doing so, co-creativity fosters creative reasoning and may improve the quality of the resulting output (Yannakakis et al., 2014). In prior work, we have defined co-creative reasoning in the context of a robot that collaborates with a human teacher to produce novel motion trajectories, while also aiming to maximize its own, partial-autonomy (Fitzgerald et al., 2017). In the context of a robot reasoning over how it may execute a task in a new environment, this co-creative process allows the robot to obtain the contextual knowledge needed to adapt its task model to meet the constraints of the novel environment.

Creative reasoning has been defined in other relevant domains, such as design creativity. Analogical reasoning is said to be a fundamental process of creativity in design (Goel, 1997). In design by analogy, a new design is created by abstracting and transferring design patterns from a familiar design to a new design problem, where the design patterns may capture relationships among the abstract function, behavior, structure, and geometry of designs. Design also entails discovery of problem constraints (Dym and Brown, 2012) including making implicit constraints in a design problem more explicit (Dabbeeru and Mukerjee, 2011). Fauconnier and Turner (2008) introduced *conceptual blending* as another process for creative reasoning. This approach addresses analogical reasoning and creativity problems by obtaining a creative result from merging two or more concepts to produce a new solution to a problem. Abstraction is enabled by mapping the merged concepts to a *generic space*, which is then grounded in the *blend space* by selecting aspects of either input solution to address each part of the problem. Applied to a robotic agent that uses this creative process to approach a new transfer problem, the robot may combine aspects of several learned tasks to produce a new behavior.

Overall, these methods for creative reasoning highlight two important components of creative reasoning: The exploration of novel solutions to a problem, and an evaluation of each candidate solution’s effectiveness. Prior work in creative reasoning (e.g., analogical reasoning, interactive co-creativity, and conceptual blending) have addressed these challenges, but not yet in the context of creative tool use by an embodied robot. This domain requires additional considerations, in that it is grounded in a robot’s action and perception (Fitzgerald et al., 2017). First, the robot has imperfect perception of its environment and/or tools, and thus may not have a complete model of the tool(s) it may use. Second, its solution must be in the form of a motion trajectory that utilizes the tool to achieve the task goals. As a result, not only is the *choice* of tool a creative one, but the *usage* of that tool is creative as well. We now review relevant literature that addresses these challenges within the robotic tool use domain.

### 2.2 Identifying Novel Tool Candidates

Existing work typically focuses on identifying the *affordances* of potential tool candidates. Affordances represent the “action possibilities” that result from the relationship between an object and its environment (Gibson, 1979). Once the affordances of candidate tools have been identified, a robot can reason over the most suitable tool for a particular task and integrate it into its motion plan (Agostini et al., 2015; Choi et al., 2018). However, identifying tool affordances is a non-trivial challenge. Recent work in computer vision has applied deep neural networks to this problem in order to visually predict the affordances for a particular tool (Do et al., 2018). The UMD Part Affordance Dataset (Myers et al., 2015) is intended to support further work on visual affordance detection. This dataset contains RGB-D images for 105 tools, grouped into 17 object categories. Each tool is photographed at roughly 75 orientations, each of which corresponds to a pixel-wise labeling according to 7 possible affordances (e.g., cutting, grasping, pounding). Other, physics-based features such as the dimensions or material of an object may also be used to judge their effectiveness as potential tools, such as when identifying a pipe as a makeshift lever to pry open a door (Levihn and Stilman, 2014). Prior work has shown that, in addition to using demonstrations to learn a task, a robot may also use demonstrations to learn to recognize the affordance-bearing subparts of a tool such that it can identify them on novel objects (Kroemer et al., 2012).

When a suitable tool replacement is not already available in the robot’s environment, it may be necessary to assemble one (Sarathy and Scheutz, 2018). Choi et al. (2018) extends the ICARUS cognitive architecture to assemble virtual tools from blocks. Nair et al. (2019) describes a method for tool construction by pairing candidate tool parts and then evaluating each pair by the suitability of the shape and attachability of the two parts. Later work (Nair and Chernova, 2020) integrates this process into a planning framework such that the task plan includes both the construction and use of the required tool.

While candidate tool identification is not the focus of this article, it is an essential step in our eventual goal of creative tool use. Overall, prior work on this topic demonstrates the task-specific requirements for identifying novel tool candidates, and the importance of identifying the salient features of a tool within the context of the current task. We now consider how these features affect the tool’s suitability when evaluating them for a particular task.

### 2.3 Evaluating Novel Tool Candidates

The shape of a tool alters its effect on its environment (Sinapov and Stoytchev, 2008), and thus a tool replacement may necessitate a change in the manipulation of that tool in order to achieve the same task goal (Brown and Sammut, 2012). For tasks involving the use of a rigid tool, the static relationship between the robot’s hand and the tooltip is sufficient for controlling the tool to complete a task (Kemp and Edsinger, 2006; Hoffmann et al., 2014). These methods assume a single tooltip for each tool, and that this tooltip is detected *via* visual or tactile means. For tasks involving multiple surfaces of the tool, the task model can be explicitly defined with respect to those segments of the tool, and repeated with tools consisting of similar segments (Gajewski et al., 2018). However, this assumes a hand-defined model that represents the task with respect to pre-defined object segments, and that these object segments are shared across tools. Given enough training examples of a task, a robot can learn a success classifier that can later be used to self-supervise learning task-oriented tool grasps and manipulation policies for unseen tools (Fang et al., 2018). We similarly aim to situate a new tool in the context of a known task, but eliminate the assumptions that 1) the new tool is within the scope of the training examples (which would exclude creative tool replacements) and 2) that the tool features relevant to the task are observable and recorded by the robot.

### 2.4 Adapting Task Models to Novel Tools

The aim of transfer learning for reinforcement learning domains is typically to use feedback obtained during exploration of a new environment in order to enable reuse of a previously learned model (Taylor and Stone, 2009). In previous work, we have shown how interaction can be used to transfer the high-level ordering of task steps to a series of new objects in a target domain (Fitzgerald et al., 2018). Similarly, the aim of one-shot learning is to quickly learn a new task, often improving learning from a single demonstration by adapting previous task knowledge. Prior work in this space focuses on learning a latent space for the task in order to account for new robot dynamics (Srinivas et al., 2018) or new task dynamics (Fu et al., 2016; Killian et al., 2017). “Meta-learning” approaches have succeeded at reusing visuomotor task policies learned from one demonstration (Chelsea et al., 2017) and using a new goal state to condition a learned task network such that it can be reused with additional task objects (Duan et al., 2017). We address the problem of a robot that has *not yet* been able to explore these relationships, aiming to enable rapid adaptation of a task model for unseen task/parameter relationships. The tool transform models learned by our approach are not specific to any task learning algorithm or representation, and thus can compliment or bootstrap methods for reinforcement, one-shot, and meta learning.

### 2.5 Summary of Related Work

Through prior work, we have identified three key steps for creative tool use: Exploring novel tools, evaluating novel tools, and adapting task models to novel tools. These stages are not entirely separable from each other, as evaluating reflects how well the robot anticipates being able to adapt its task model for a particular tool, and exploration results in a set of tools that meet some criteria such that they may be evaluated in the context of the task. A common theme through all three steps is the importance of *constraints* (e.g., tool shape, segments, or visual features) that dictate how a task model may be adapted to a particular tool, and as a result, play a role in the exploration and evaluation steps as well.

In the rest of this paper, we focus on this challenge of identifying and modeling *constraints*, and demonstrate how these constraints may be used in the evaluating and adapting steps of creative tool use. While we do not explicitly address creative tool exploration, we aim for this work to support future research on identifying these constraints visually to enable this exploration.

## 3 Tooltips as Constraints

Suppose that a robot has learned a trajectory $T_{a}=\left[p_{a}^{\left(0\right)},p_{a}^{\left(1\right)},\ldots ,p_{a}^{\left(n\right)}\right]$ consisting of end-effector poses $p_{a}^{\left(i\right)}$ for a particular task using tool *a*, and now must complete the same task using a different tool *b*. Our goal is to transform each pose individually for tool *b*. Representing an original pose for tool *a* in terms of its 3 × 1 translational vector **t**_**a**_ and 4 × 1 rotational vector **r**_**a**_, we transform it into a pose **p**_**b**_ for tool *b* as follows:

$$p_{b}=\phi_{a}^{b}\left(p_{a}\right)=\left\langle t_{a}+\hat{t},r_{a}\cdot \hat{r}\right\rangle$$ (1)

Here, $r_{a}\cdot \hat{r}$ refers to the Hamilton product between the two quaternions. This definition relies on a known transform between tools *a* and *b*, which requires knowledge of the appropriate “reference” point for both tools such that their transform can be computed. Neither reference point is initially known by the robot, however, nor can it be extracted from the trajectory which is represented according to the robot’s end-effector, and not according to any point on the tool itself.

**FIGURE 1:**
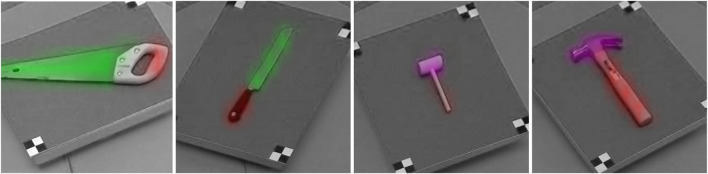
Affordance regions may be broad, spanning multiple possible tooltips. As a result, predicting the affordance region is not sufficient to plan with respect to that tool’s tooltip. For example, the full blade surfaces of the saw and knife are labeled as enabling the “cutting” affordance (highlighted in green) and the “grasping” affordance (highlighted in red); however, cutting is only performed using the edge of the blade, and requires that the blade be oriented toward the cutting target. Similarly, different points of a hammer head may enable different tasks (e.g., pounding versus prying), and thus detecting a task-independent affordance region (highlighted in purple) is not sufficient to plan a task trajectory.

Identifying the “reference point” for a tool is non-trivial. While prior work has addressed the problem of identifying affordance regions of a tool, these regions are too broad to characterize the transform between two tools. Figure 1 illustrates examples of these labeled affordance regions based on the UMD Part Affordance Dataset (Myers et al., 2015). While this dataset is relevant to identifying similar regions on two separate tools, it does not address the problem of specifying the equivalent points of a tool that may be used to transform the trajectory for a *particular task* from one tool to another. For example, the full blade of a knife may be labeled as enabling the “cutting” affordance (Figure 1), even though a cutting task is likely to be performed with respect to only the edge of the blade. Furthermore, since affordance data is presented in the form of pixel-wise image labels, it does not provide any data concerning the kinematic implications of using this tool. Since the tool is observed and labeled from a static, overhead perspective, affordance data is only available along a single 2D plane, and thus does not indicate the *orientation* at which each affordance is or is not valid.

This is essential for manipulating the tool properly; even if the robot were to determine that the relevant surface of a knife is located along the edge of its blade, the blade must still be oriented carefully with respect to the cutting target for the task to be completed successfully. We refer to the acting surface of the tool (e.g., a singular point along the edge of the knife blade, or a singular point on a mallet’s pounding surface) as a **tooltip** that is defined by a pose containing both the position and orientation of that tooltip. In summary, we expect that **successful task completion relies on the robot having a model of the composite transform between 1) the end-effector, 2) its grasp of the tool** (highlighted in red in Figure 1), **and 3) the tooltip position and orientation**.

While we may mathematically represent a tooltip as a singular pose, practically, however, there are likely many possible tooltips that may lead to successful task execution. Additionally, the constraint over the tooltip may also differ depending on the context in which it is used: The orientation of a hammer is constrained along two axes when hammering a nail, but the hammer may still be rotated around the nail (e.g., its “yaw” rotation) without affecting task performance. This example supports the notion of a *one-to-many* relationship between 1) a tooltip and 2) the tool poses that enable that tooltip to be used.

In the remainder of this paper, we explore this one-to-many relationship. In Section 4, we demonstrate how a single tooltip can be expanded into a set of effective tool poses, thus highlighting the challenges of learning tooltip constraints. In Section 5, we consider this relationship in the opposite direction, and present two models for deriving a single tooltip from a set of valid poses demonstrated by a human teacher.

## 4 Characterizing Tool Constraints

We first explore the effect of tooltip constraints by expanding a single tooltip into a set of tool poses that result in successful task execution. To do so, we transform a trajectory that results in successful task execution (and thus the tooltip is implicitly-defined) such that the tooltip’s trajectory is perturbed slightly. In doing so, we can evaluate the effect of that perturbation on task performance, and ultimately model the constraints that dictate which poses result in successful use of the tooltip.

In this section, we address two key research questions:

1) How do changes in tool pose affect task performance?
2) How do the constraints on tool pose differ across tools and/or tasks?

### 4.1 Evaluating Tool-Task Constraints in Simulation

**FIGURE 2:**
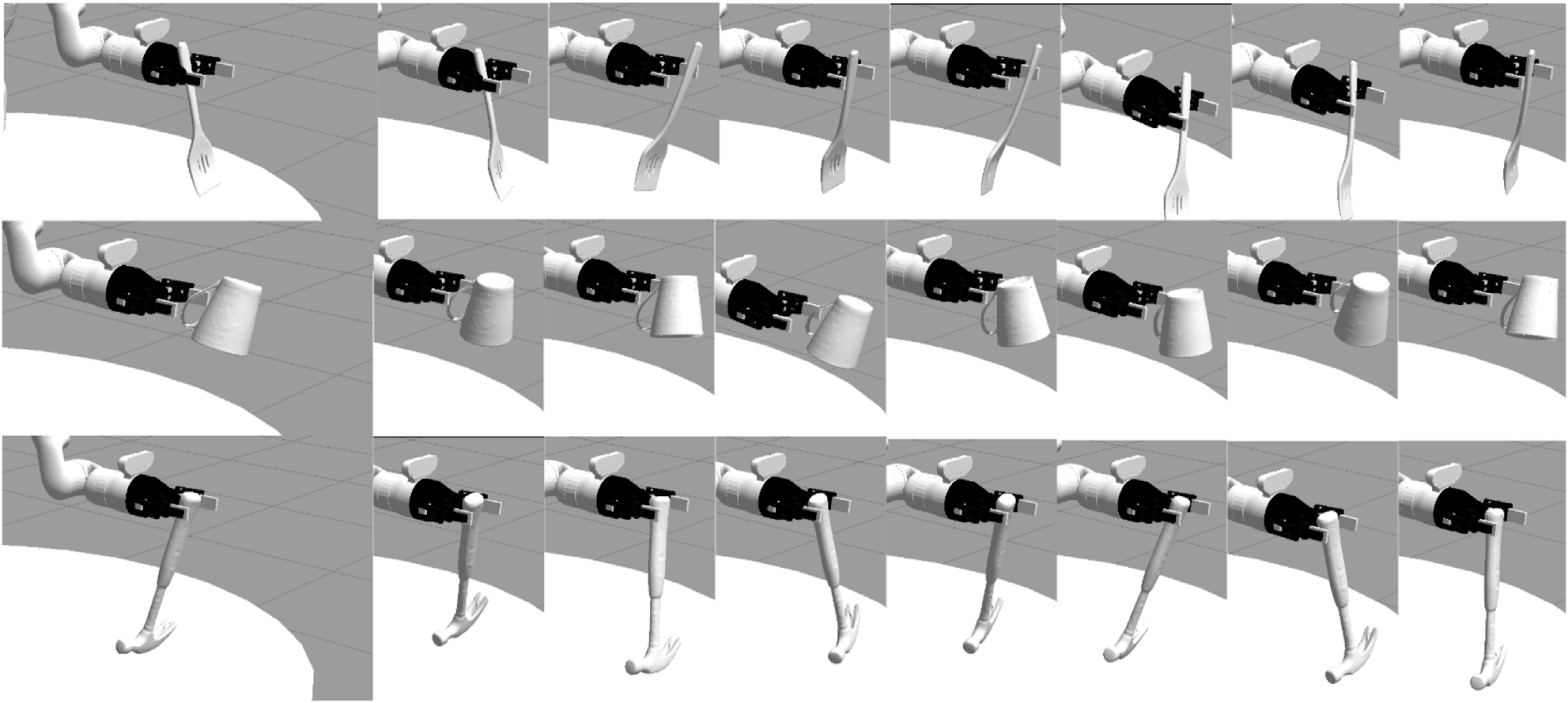
We performed an evaluation across three tools: a spatula, mug, and hammer. For each tool, we perturbed the trajectory of the tooltip by adjusting the robot’s grasp of the tool. These pose variations are just a small set of the 729 perturbations we evaluated for each tool-task pairing.

We address these research questions by evaluating the performance of a large set of trajectory perturbations using a simulated 7-DOF Kinova Gen3 robot arm situated on a round table in a Gazebo simulated environment. We evaluated the effect of trajectory perturbations on three tools: A hammer, a mug, and a spatula (Figure 2). We fixed the robot’s grasp as a static transform between the robot’s gripper and the tool, and thus did not evaluate the effects of the robot’s grasp strength or stability on tool use.

**FIGURE 3:**
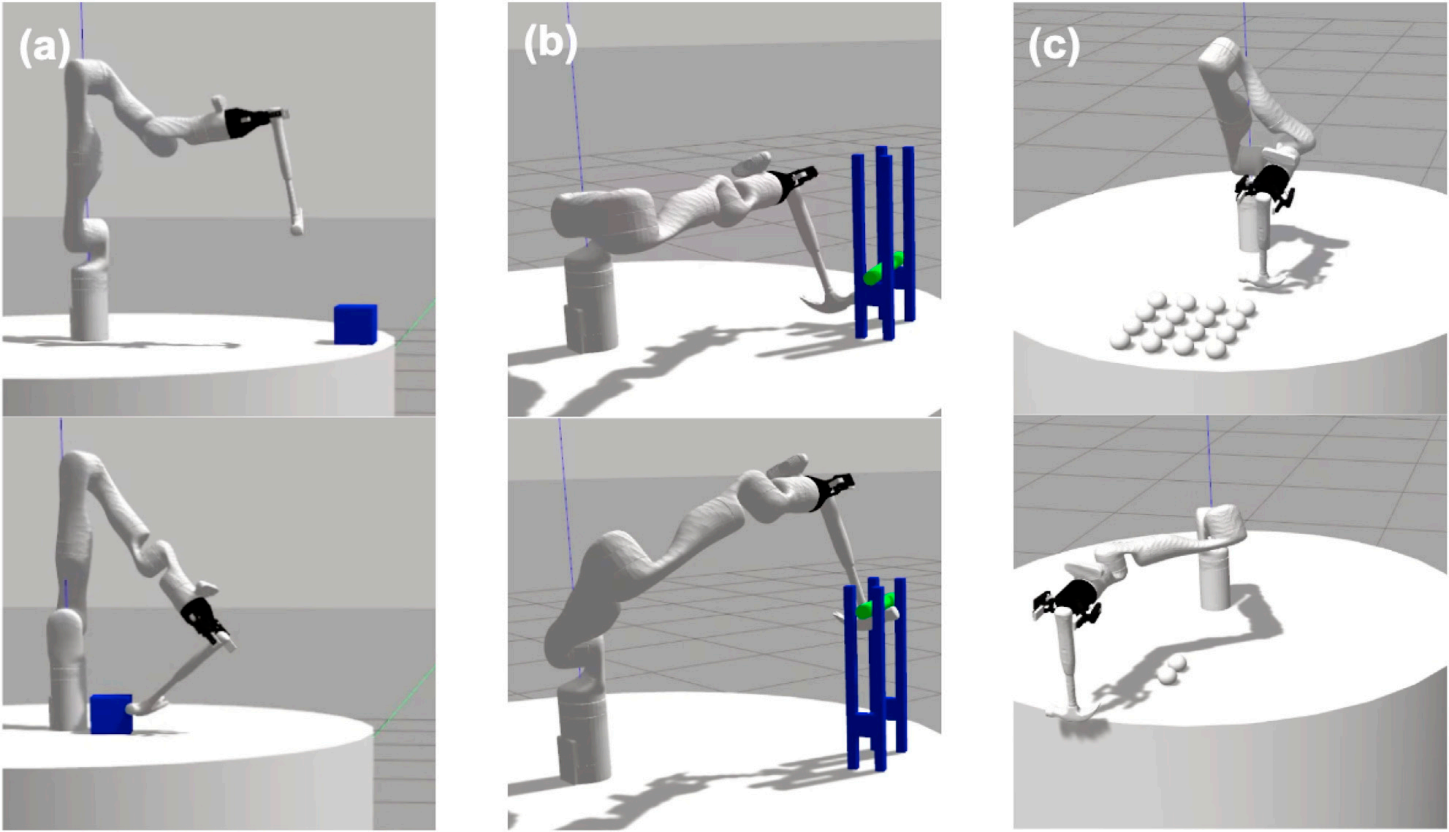
Initial and goal states for the **(A)** hooking, **(B)** lifting, and **(C)** sweeping tasks.

For each tool, we provided a demonstration of three tasks: Hooking (Figure 3A), lifting (Figure 3B), and sweeping (Figure 3C). Each demonstration was provided in a Gazebo simulator as a set of end-effector keyframes. Depending on the tool being demonstrated, this resulted in 5-7 keyframes for hooking, 4-6 for lifting, and 13-18 for sweeping. These end-effector keyframes were then converted to keyframe trajectories represented in the robot’s joint-space. We used the MoveIt (Coleman et al., 2014) implementation of the RRTConnect planner to plan between joint poses during trajectory execution. We simulated a trajectory perturbation by altering the rigid transform between the robot’s gripper and the tool itself, according to a pre-determined set of position and orientation alterations that are consistent across all tools and tasks. As a result, each trajectory perturbation is identical with respect to the robot’s end-effector, but differ with respect to the trajectory of the tool itself. This allowed us to use the same joint-space trajectory for all perturbations of a single tool-task pairing, thus reducing the likelihood of planning errors across all perturbations and also minimizing any changes in the robot’s joint motion that might affect task performance. Despite the same trajectory being executed across all perturbations of a single tool-task pairing, planning errors may still occur when a perturbation results in the tool colliding with its environment, thus preventing the rest of the trajectory from being executed.

Each perturbation resulted from a unique permutation of changes applied to the tool’s demonstrated position along the *x*, *y*, and *z* axes and demonstrated orientation along the roll, pitch, and yaw axes. The tool’s *x*, *y*, and *z* positions were each configured at one of three distances from the demonstrated tool position: [ − 0.01, 0, 0.01] meters. The tool’s roll, pitch, and yaw rotations were each configured at one of three angles from the demonstrated tool orientation: $\left[-\frac{\pi }{16},0,\frac{\pi }{16}\right]$ radians. These position and orientation perturbations were empirically chosen such that, when combined, their effect on task performance can be observed on a spectrum. We observed that larger ranges of pose or orientation changes would be less likely to result in completion of any aspect of the task, whereas smaller ranges may not fully explore the range of successful perturbations. However, as we note later in Section 4.3, we observe that different tools vary in their sensitivity to these perturbations, and thus a more fine-grained set of perturbations should be explored in future work.

Overall, the permutation of these configurations resulted in a total of 3^6^ = 729 perturbations for each tool-task pairing. We executed each perturbation twice in simulation (to account for the non-deterministic effects of the simulator dynamics) and recorded the average performance of the two trials, with performance being measured according to task-specific measures. All performance metrics were scaled to a 0–1 range. In the hooking task, performance was measured as the distance (in meters) between the box and the robot’s base, with less distance correlating to higher performance. The initial and goal states of this task are shown in Figure 3A. In the lifting task, the robot’s performance was measured as the green bar’s height above the table (in meters). A small number of trials resulted in the bar being removed from the support structure entirely. In these cases, we recorded the performance as that of the task’s initial state (i.e., a failure case). Figure 3B shows the initial and goal states of this task. In the sweeping task, performance is measured as the number of spheres that were swept off the table, with maximum performance being 16 spheres. The initial and goal states of this task are shown in Figure 3C.

### 4.2 Results

**FIGURE 4:**
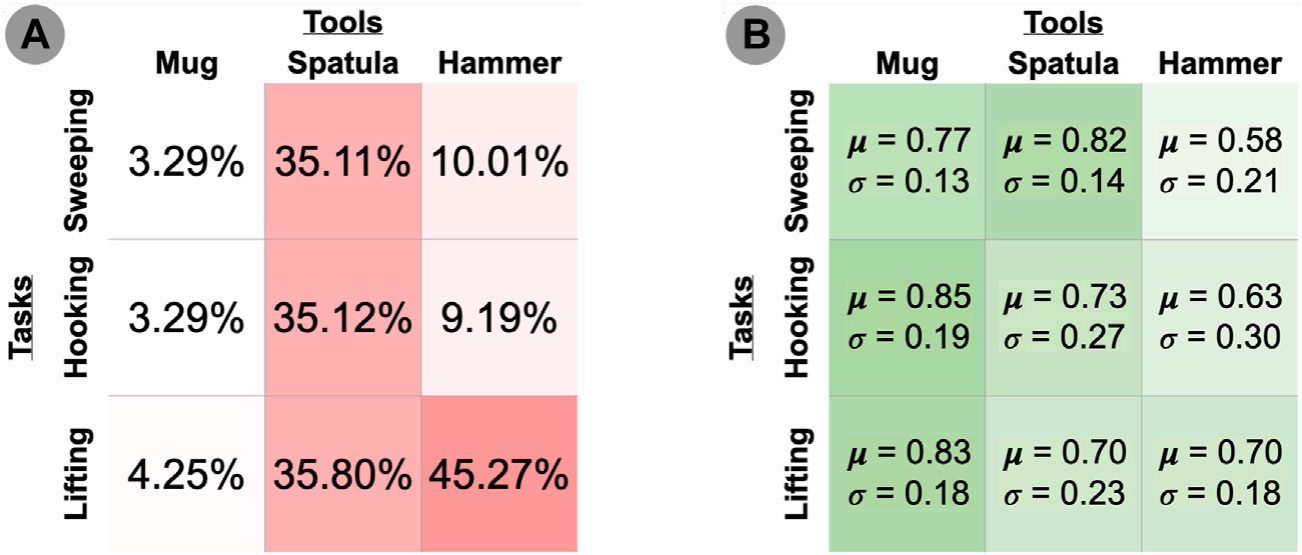
**(A)** Percentage of failed trials (performance ≤ 0.05). Darker cells indicate higher percentage of failed trials. **(B)** Mean and standard deviation performance of thresholded (performance > 0.05) trials. Darker cells indicate higher mean performance.

Our evaluation measured how sensitive each tool-task pairing is to perturbations of the tooltip’s trajectory: The more sensitive the tool-task pairing is to perturbations, the more likely that a perturbation will lead to a task failure. Low task performance may be caused by the tooltip no longer contacting any relevant objects in the task (and thus leaving the task in its initial state), or by collisions between the tool’s new configuration and its environment that prevent the robot from executing the full trajectory. We set a threshold performance of 0.05 (on a 0–1 scale), and report the percentage of perturbations that fail to exceed this threshold in Figure 4.

**FIGURE 5:**
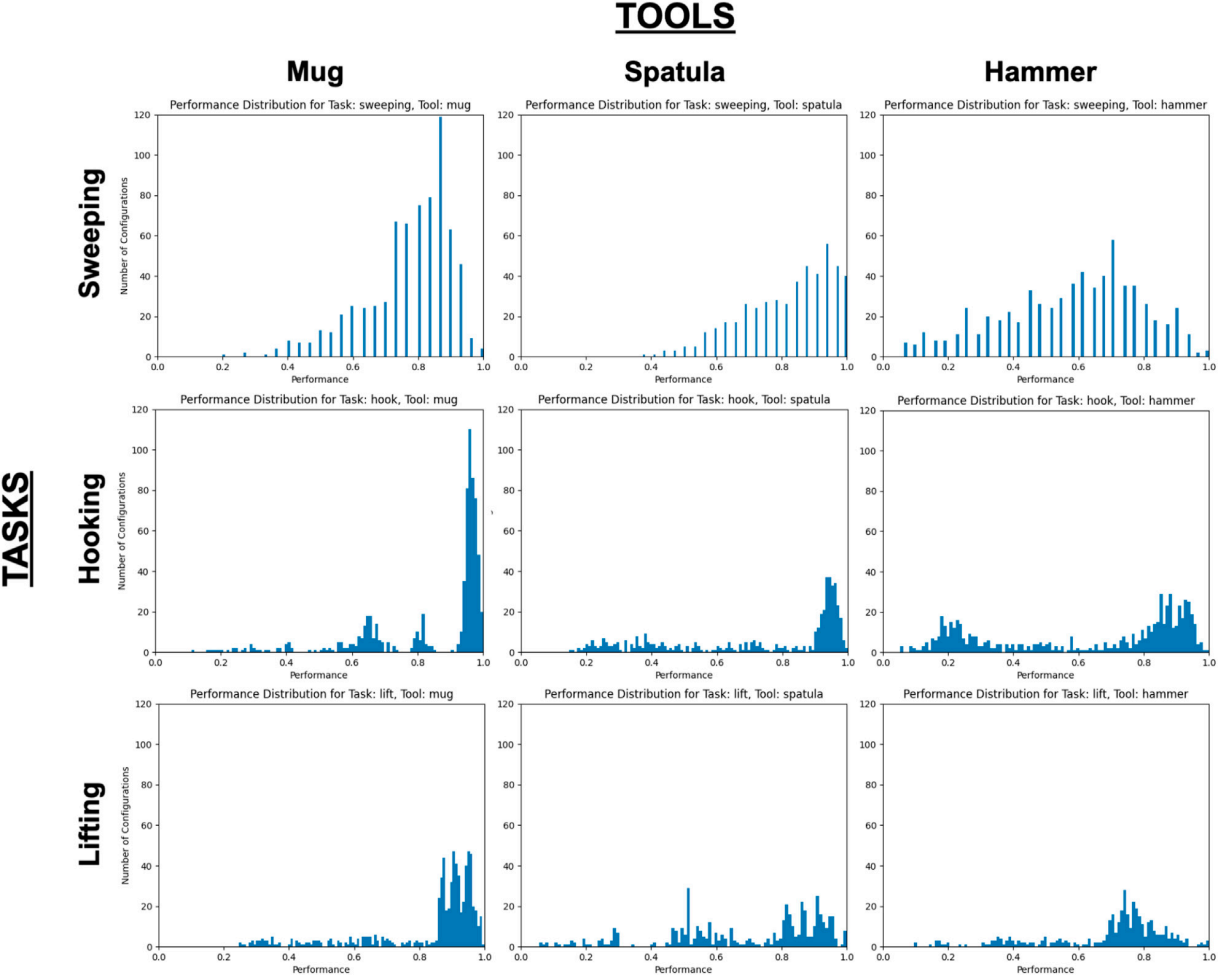
Performance distributions over all tool-task pairings, with all trials with performance ≤ 0.05 excluded. *X*- and *Y*-axes are consistent across all graphs.

We include only the set of perturbations that exceed this threshold in the histograms in Figure 5, which illustrate the performance distributions over the set of perturbations exceeding this threshold. Since the original, unperturbed pose is already known to achieve near-optimal task performance, these graphs illustrate how many perturbations of that original pose still fulfill the tooltip constraints and result in high performance (i.e., the perturbations resulting in the peak observed near *x* = 1.0 on each graph). We report the mean and variance over these performance results in (Figure 4B).

**FIGURE 6:**
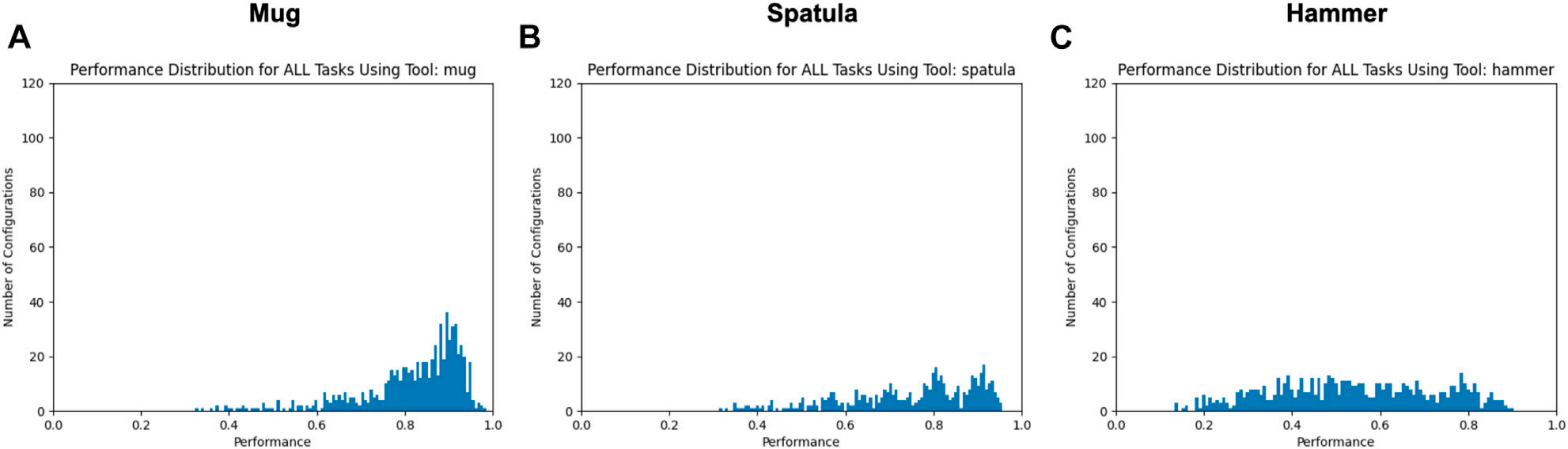
Mean performance distributions using each tool for all tasks, with all trials with mean performance ≤ 0.05 excluded. *X*- and *Y*-axes are consistent across all graphs.

Figure 6 shows the distribution over the *mean performance* over all three tasks; that is, the performance metric for each perturbation is the average of its performance on the sweeping, hooking, and lifting tasks. We again only consider datapoints above a performance threshold > 0.05 in order to focus on the set of *valid* tooltip constraints for each tool.

### 4.3 Discussion

*Research Question #1: How do changes in tool pose affect task performance?* **The relationship between performance and tool pose may be non-linear.** If this relationship were linear, we would expect Figure 5 to primarily contain Gaussian-like performance distributions, such that as the robot evaluates trajectory perturbations further from the original trajectory, its performance resulting from those perturbations decreases proportionally. While this is the case in some tool-task pairings (e.g., all tools used for the sweeping task, and the lifting task using the hammer), other performance distributions appear to be bi-modal in nature (e.g., using the hammer in the hooking task or using the spatula for lifting) or contain several peaks (e.g., using the mug for hooking). This suggests that there is a non-linear relationship between changes in the tool pose, and its resulting effects on task performance. Note that in our evaluation, we applied trajectory perturbations according to the single tooltip that was demonstrated for each tool-task pairing. An opportunity for future research is the identification of alternate tooltips based on the tool’s shape or structure.

*Research Question #2: How do the constraints on tool pose differ across tools and/or tasks?* **Tools differed in their sensitivity to pose changes.** For example, using the spatula tool resulted in the highest percentage of failed trials (35.11–35.8%) across all three tasks, while the mug resulted in the lowest (3.29–4.25%) across all three tasks. One hypothesis for this performance difference is that since the mug was the smallest tool, changes in the tool pose had a smaller effect on its *tooltip* pose in comparison to the taller tools (spatula and hammer). We observed widely varying failure rates when using the hammer, ranging from 9.19 to 10.01% on the hooking and sweeping tasks, respectively, and 45.27% on the lifting task. One reason for this performance difference may be that a different tooltip was used for the lifting task compared to the hooking and sweeping tasks. In the former, the robot uses a “corner” of the hammer to lift the bar (Figure 3B), whereas the hooking and sweeping tasks use a wider surface area of the hammer as a tooltip. This may provide more tolerance to pose perturbations. **Overall, this suggests that the sensitivity of tooltip constraints depends on the surface of the tool being used.**

Figure 6 also supports this hypothesis. These distribution graphs reflect the consistency in tooltip constraints across tasks. While the geometry of the tool itself remains constant across tasks, the same tooltip is not necessarily used across tasks (e.g., using separate surfaces of the hammer for sweeping vs lifting). The reduced performance shown in these graphs (in comparison to Figure 5) indicates that **the tooltip constraints applied to one task may not be generalizable to other tasks using the same tool.**

We now consider the challenge of how a robot may quickly learn these constraints in the context of a new tool, and whether we can model the instances in which a robot can reuse a learned tooltip model in the context of another task. While a robot can learn to use a tool through demonstrations, the one-to-many mapping between tooltip constraints and the set of tool poses that meet those constraints means that there are many possible demonstrations that a robot may receive for a tool/task pairing. Learning the underlying tool constraint is therefore a challenge, as the teacher is providing demonstrations that sample from an unknown, underlying relationship between the end-effector and the tooltip. In the next section, we explore how a robot can utilize corrections in order to model and learn the underlying tooltip constraint.

## 5 Learning Constraints From Interactive Corrections

In the previous section, we evaluated the one-to-many mapping between tooltips constraints and end-effector poses that meet those constraints. In order to adapt the robot’s task model to a novel tool, however, we also need to analyze this mapping in the reverse direction: inferring the underlying tooltip constraint that has resulted in a set of corresponding end-effector poses.

We address this challenge in the context of a robot that learns from demonstrations by a human teacher who is familiar with the task and tool that the robot aims to use. By comparing two trajectories, each using a separate tool to complete the same task, we aim to model the relationship between the two tooltips constraints such that it can be reused in the context of another task.

While a robot can quickly receive demonstrations (Argall et al., 2009; Chernova and Thomaz, 2014) using a new tool, these demonstrations may not be sufficient to learn the underlying tooltip constraints. Due to the unstructured nature of task demonstrations, the two demonstrations (each provided using a different tool) may vary in ways that do not reflect how the task should be adapted based on which tool is used. For example, the teacher may choose a different strategy for completing the task with the second tool, or the robot may be starting from a new arm configuration when the teacher demonstrates the task with the second tool. For these reasons, we utilize *corrections* of the robot’s behavior, which have been shown to be effective interface for adapting a previously-learned task model (Argall et al., 2010; Sauser et al., 2012; Bajcsy et al., 2018). Rather than have the teacher provide a new demonstration using the new tool, the robot attempts to complete the task on its own and is interrupted and corrected by the teacher throughout its motion. As a result, this interaction results in a series of correction pairs, where each pair represents the robot’s originally-intended end-effector pose and its corresponding, corrected pose that was indicated by the teacher.

Our research questions are as follows:

1) How can we model a tooltip constraint using data provided *via* sparse, noisy corrections?
2) Under what conditions can the tooltip constraints learned from corrections on one task be used to adapt other task models to the same replacement tool? What characteristics of the tool and task predict whether a previously-learned tooltip constraint can be applied?

In the following sections, we address these research questions using the Transfer by Correction algorithm, which we first described in Fitzgerald et al. (2019).

### 5.1 Problem Definition

We assume that each demonstration consists of a series of keyframes (Akgun et al., 2012). The robot receives corrections by executing a trajectory planned using the original task model, pausing after a time interval defined by the keyframe timings set during the original demonstration. The teacher then moves the robot’s gripper to the correct position, after which the robot resumes task execution for the next time interval, repeating the correction process until the entire task is complete. Each resulting correction at interval *i* consists of the original pose $C_{a}^{i}$ (using tool *a*) and the corrected pose $C_{b}^{i}$ (using new tool *b*) at keyframe *i*. A collection of *K* corrections (one for each of *K* keyframes) results in a *K* x 2 correction matrix:

C=Ca0Cb0Ca1Cb1…CaKCbK (2)

Each corrected pose $C_{b}^{i}$ provides a sample of the transfer function value with the original pose $C_{a}^{i}$ at keyframe *i* as input, plus some amount of error from the optimal correction pose:

$$C_{b}^{i}=\phi_{a}^{b}\left(C_{a}^{i}\right)+\epsilon \;\epsilon_{n}∼N\left(0,\;\sigma_{n}^{2}\right)$$ (3)

We assume ϵ is sampled from a Gaussian noise model for each axis *n* ∈ [1…6] of the 6D end-effector pose. Our aim is to learn a transfer function *ϕ* that optimally reflects the tooltip constraints, using a correction matrix **C**.

### 5.2 Approach: Transfer by Correction

Given a task trajectory **T** for tool *a* consisting of a series of *t* poses in task space such that **T** = [**p**_**0**_, **p**_**1**_, *…*, **p**_**t**_], we transform each pose individually for tool *b*. Representing an original pose for tool *a* in terms of its 3 × 1 translational vector **t**_**a**_ and 4 × 1 rotational vector **r**_**a**_, we transform it into a pose **p**_**b**_ for tool *b* as follows:

$$p_{b}=\phi_{a}^{b}\left(p_{a}\right)=\left\langle t_{a}+\hat{t},r_{a}\cdot \hat{r}\right\rangle$$ (4)

Here, $r_{a}\cdot \hat{r}$ refers to the Hamilton product between the two quaternions. The goal is now to estimate the optimal rotational $\hat{r}$ and translational $\hat{t}$ transformation components from the corrections matrix **C**, and then apply these transformations to the trajectory **T**. Our approach addresses this goal by (1) modeling **C**, particularly the relationship between each correction’s translational and rotational components, 2) sampling a typical translational transformation $\hat{t}$ and rotational transformation $\hat{r}$ from this transform model, and 3) applying $\hat{t}$ and $\hat{r}$ to transform each pose in the task trajectory according to Equation 4.

### 5.3 Task Constraints

**FIGURE 7:**
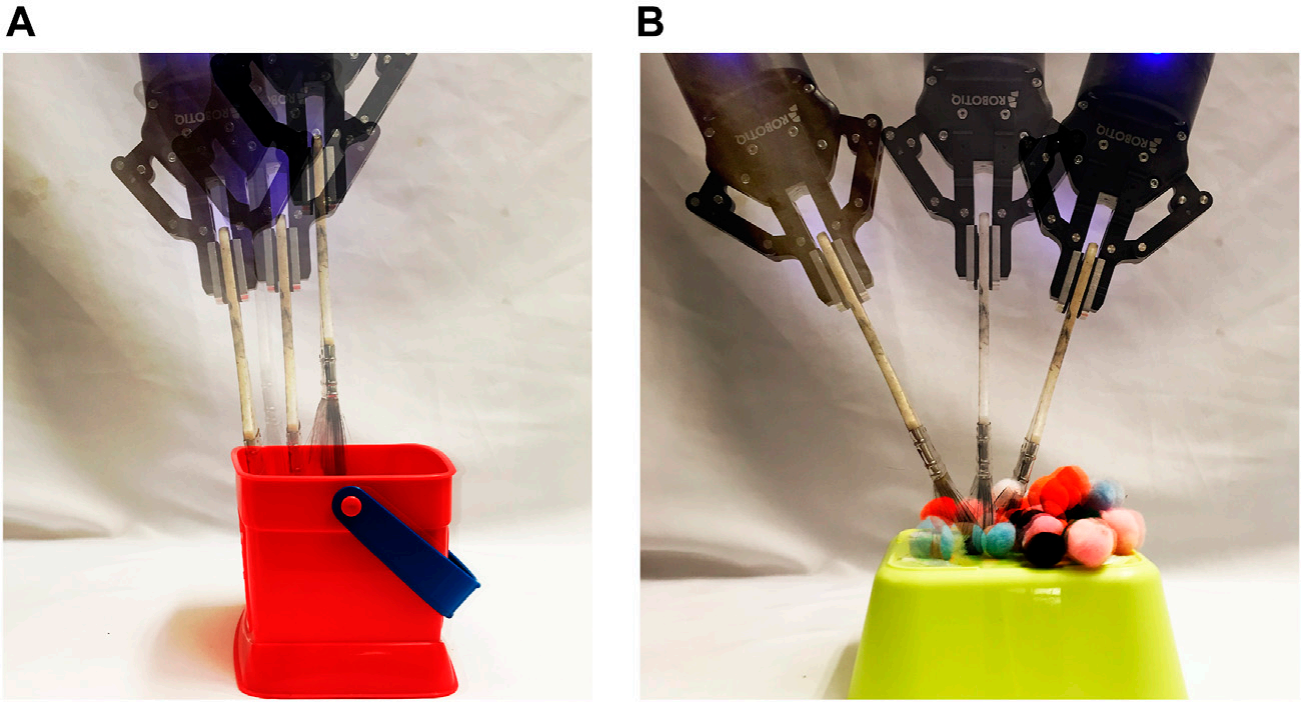
Poses meeting the same orientation constraint share similar orientations but vary more in their position **(A)**, whereas poses meeting the same center-of-rotation constraint rotate around the tooltip **(B)**.

We observe that corrections indicate constraints of the tooltip’s position and/or orientation, and that these constraints are reflected in the relationship between the translation and rotation components of each correction. Broadly, each correction may primarily indicate:

• An *unconstrained* point in the trajectory, and thus should be omitted from the tool transform model.
• An *orientation constraint*, where the rotation of the tooltip (and thus the end effector) is constrained more than its position (e.g., hooking a box is constrained more by the orientation of the hook than its position, as in the left of Figure 7).
• A *center-of-rotation constraint*, where the position of the tooltip is constrained more than its rotation (e.g., sweeping a surface with a brush). Note that the *tooltip* position is the center of this constraint rather than the end-effector itself, and thus the range of valid end-effector poses forms an arc around the tooltip, and its orientation remains angled toward the tooltip (e.g., Figure 7B).

We define two *tool transform models*, first presented in Fitzgerald et al. (2019), each reflecting either orientation or center-of-rotation constraints. We fit the corrections matrix to each tool transform model, using RANSAC (Fischler and Bolles, 1981) to iteratively estimate the parameters of each model while discarding outlier and unconstrained correction data points. Each iteration involves 1) Fitting parameter values to a sample of *n* datapoints, 2) Identifying a set of inlier points that also fit those model parameters within an error bound of *ϵ*, and 3) Storing the parameter values if the inlier set represents a ratio of the dataset > *d*. The RANSAC algorithm relies on a method for fitting parameters to the sample data, and a distance metric for a datapoint based on the model parameters. These are not defined by the RANSAC algorithm, and so we specify the parameterization and distance metric according to the tool transform model used, which we describe more in the following sections. We define an additional method to convert the best-fitting parameters following RANSAC completion into a typical transform that can be applied to poses.

### 5.4 Linear Tool Transform Model

**FIGURE 8:**
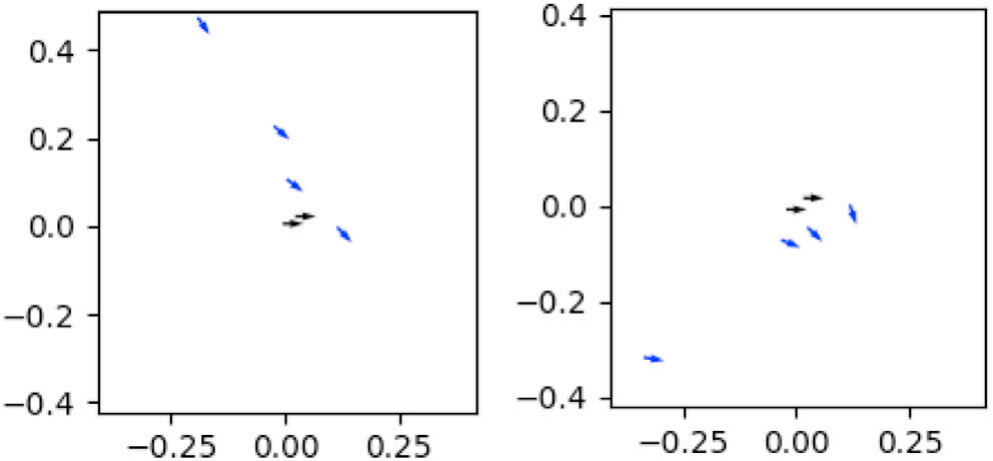
Each plot represents one set of corrections for a task. The position of each arrow represents the change in < *x*, *y* > position, and points in the direction of the change in orientation introduced by that correction. Orientation constraints can be seen in **(A)**, where the majority of corrections on this tool have low variance in their orientation, but higher variance in their *x*-*y* position. Center-of-rotation constraints can be seen in **(B)**, where the majority of corrections arc around a singular center of rotation, and orientation is dependent on the *x*-*y* position. Unconstrained keyframes (colored grey) are located near (0,0).

Based on the *orientation* constraint type, we first consider a linear model for correction data, where corrections fitting this model share a linear relationship between the translational components of the corrections, while maintaining a constant relationship between the rotational components of corrections (Figure 8A). We model this linear relationship as a series of coefficients obtained by applying PCA to reduce the 3D position corrections to a 1D space.

#### 5.4.1 RANSAC Algorithm Parameters

The RANSAC algorithm is performed for *k* iterations, where we use the estimation

$$k=\frac{\log\left(1.0-p\right)}{\log\left.\left(1.0-w^{n}\right)\right)}$$ (5)

with desired confidence *p* = 0.99 and estimated inlier ratio *w* = 0.5. Additional parameters are as follows: *n* = 2 is the number of data points sampled at each RANSAC iteration, *ϵ* = 0.01 is the error threshold used to determine whether a data point fits the model, and *d* = 0.5 is the minimum ratio between inlier and outlier data points in order for the model to be retained.

#### 5.4.2 Model Parameter Fitting

Model fitting during each iteration of RANSAC consists of reducing the datapoints to a 1D model using PCA, returning the mean translational correction and the coefficients for the first principal component of the sample **S**:

$$\Theta_{\text{linear}}\left(S\right)=\left\langle \theta_{\mu },\theta_{u}\right\rangle \;\theta_{\mu }=\frac{1}{\left|S\right|}\underset{p\in S}{\sum }p_{t}$$ (6)

where **p**_**t**_ is the 3 × 1 translational difference indicated by the correction **p**, **S** is the subset of the corrections matrix **C** sampled during one iteration of RANSAC such that **S** ⊂ **C**, and *θ*_**u**_ is the eigenvector corresponding to the largest eigenvalue of the covariance matrix $\Sigma =\frac{1}{\left|S\right|}S_{t}^{T}S_{t}$ .

#### 5.4.3 Error Function

Each iteration of RANSAC calculates the total error over all data points fitting that iteration’s model parameters. We define the error of a single correction datapoint **p** as the sum of its reconstruction error and difference from the average orientation correction, given the current model parameters *θ*:

$$\delta_{\text{linear}}\left(p,\theta \right)=\|p_{t}-\left(\theta_{\mu }+{\left(p_{t}-\theta_{\mu }\right)}^{T}\theta_{u}{\theta_{u}^{T}}^{+}\right)\|+\gamma \left({\left(1-{\bar{q}}_{n}p_{n}^{T}\right)}^{2}\right)$$ (7)

where **x** ^+^ indicates the Moore-Penrose pseudo-inverse of a vector, **p**_**n**_ is the unit vector representing the orientation difference indicated by the correction **p**, ${\bar{q}}_{n}$ is a unit vector in the direction of the average rotation sampled from the model (defined in the next section), and *γ* is the weight assigned to rotational error (*γ* = 1 in our evaluations).

#### 5.4.4 Sampling Function

After RANSAC returns the optimal model parameters and corresponding set of inlier points $\hat{I}\subset C$ , the rotation and translation components of the transformation are sampled from the model. We define the sampling function according to the estimated “average” rotation $\bar{q}$ :

$$\Psi {\left(\hat{I},\hat{\theta }\right)}_{\text{linear}}=\left\langle \bar{q},\bar{t}\right\rangle \;\bar{q}=\underset{q\in S^{3}}{arg\;max}q^{T}Mq\;M=\frac{1}{\left|\hat{I}\right|}\underset{p\in \hat{I}}{\sum }p_{q}^{i}{p_{q}^{i}}^{T}$$ (8)

The solution to $\bar{q}$ for this maximization problem is the eigenvector corresponding to the largest eigenvalue of *M* (Markley et al., 2007). The sample translation $\bar{t}$ is the 3D offset corresponding to the mean value $\bar{z}$ from the 1D projection space:

$$\bar{t}={\hat{\theta }}_{\mu }+\bar{z}{{\hat{\theta }}_{u}}^{T+}\;\bar{z}=\frac{1}{\left|\hat{I}\right|}\underset{p\in \hat{I}}{\sum }{\left(p_{t}-{\hat{\theta }}_{\mu }\right)}^{T}{\hat{\theta }}_{u}$$ (9)

### 5.5 Rotational Tool Transform Model

We now consider a model for corrections reflecting a *center-of-rotation constraint*, in which we make the assumption that corrections indicate a constraint over the tool tip’s *position*. Since the tool tip is offset from the end-effector, the position and rotation of the end-effector are constrained by each other such that the end-effector revolves around the tool tip (Figure 8B). We model this relationship by identifying a center-of-rotation (and corresponding rotation radius) for the tool tip, from which we can sample a valid end-effector position and rotation.

#### 5.5.1 RANSAC Algorithm Parameters

We use the same parameters for *k*, *w*, *d* as in the linear model. We sample *n* = 3 points at each iteration, and use the error threshold *ϵ* = 0.25. We define functions for model parameterization, error metrics, sampling, and variance in the following sections.

#### 5.5.2 Model Parameter Fitting

We define the optimal model parameters for each iteration of RANSAC as the center-of-rotation (and corresponding rotation radius) of that iteration’s samples **S**:

$$\Theta_{\text{rotation}}\left(S\right)=\left\langle \theta_{c},\theta_{r}\right\rangle$$ (10)

where *θ*_**c**_ is the position of the center-of-rotation that minimizes its distance from the intersection of lines produced from the position and orientation of each correction sample:

$$\theta_{c}=\underset{c}{arg\;min}\overset{\left|S\right|}{\underset{i=1}{\sum }}D{\left(c;a_{i},n_{i}\right)}^{2}$$ (11)

where **a**_**i**_ and **n**_**i**_ are the position and unit direction vectors, respectively, for sample *i* in **S**:

$$a_{i}={\left[x_{i},y_{i},z_{i}\right]}^{T}\;n_{i}=\left.\left(q_{i}\cdot {\left[0,1,0,0\right]}^{T}\right)\right)\cdot q^{\prime }$$ (12)

Here, **q**_**1**_ ⋅ **q**_**2**_ refers to the Hamilton product between two quaternions, and **q**′ is the inverse of the quaternion **q**:

$$q^{\prime }={\left[w,x,y,z\right]}^{\prime T}={\left[w,-x,-y,-z\right]}^{T}$$ (13)

We solve for the center-of-rotation by adapting a method for identifying the least-squares intersection of lines Traa (2013). We consider each sample *i* to be a ray originating at the point **a**_**i**_ and pointing in the direction of **n**_**i**_. The center-of-rotation of a set of these rays is thus the point that minimizes the distance between itself and each ray. We define this distance as the piecewise function:

$$D\left(c;a,n\right)=\left\{\begin{array}{cc} \|\left(c-a\right)-d\cdot n{\|}_{2} & \text{if }d>0\\ \|c-a{\|}_{2} & \text{otherwise} \end{array}\right.$$ (14)

where *d* is the distance between **a** and the projection of the candidate centerpoint **c** on the ray:

$$d={\left(c-a\right)}^{T}n$$ (15)

We solve for *θ*_**c**_ using the SciPy implementation of the Levenberg-Marquardt method for non-linear least-squares optimization, supplying Equation 14 as the cost function. We then solve for the radius corresponding to *θ*_**c**_:

$$\theta_{r}=\frac{1}{\left|S\right|}\overset{\left|S\right|}{\underset{i=0}{\sum }}\|a_{i}-\theta_{c}\|$$ (16)

#### 5.5.3 Error Function

We define the error of a single data point **p** as its distance from the current iteration’s center-of-rotation estimate:

$$\delta_{\text{rotation}}\left(p,\theta \right)={\left(\frac{D\left(c;a_{p},n_{p}\right)}{d_{p}}\right)}^{2}$$ (17)

Where *d*_*p*_ is defined in Equation 15.

#### 5.5.4 Sampling Function

After RANSAC returns the optimal model parameters and corresponding set of inlier points $\hat{I}\subset C$ , the rotation component of the transformation is first sampled using the “average” rotation ${\bar{q}}_{c}$ from ${\hat{\theta }}_{c}$ to all inlier points:

$${\bar{q}}_{c}=\underset{q\in S^{3}}{arg\;max}q^{T}Mq\;M=\frac{1}{\left|\hat{I}\right|}\underset{p\in \hat{I}}{\sum }r_{p}r_{p}^{T}$$ (18)

Where **r**_**p**_ is the quaternion rotation between ${\hat{\theta }}_{c}$ and the position of **p**, defined by normalizing the quaternion consisting of the scalar and vector parts:

$$r_{p}=\left\langle \|a{\|}^{2}+ba^{T},b^{T}\times a\right\rangle$$ (19)

$$a=p_{t}-{\hat{\theta }}_{c}\;b=\left[\|a\|,0,0\right]$$ (20)

The optimal ${\bar{q}}_{c}$ is the eigenvector corresponding to the largest eigenvalue of *M*; this represents the sampled rotation from ${\hat{\theta }}_{c}$ .

We then sample $\bar{t}$ by projecting the point at distance ${\hat{\theta }}_{r}$ from ${\hat{\theta }}_{c}$ in the direction of ${\bar{q}}_{c}$ :

$$\bar{t}={\hat{\theta }}_{c}+{\left[\left({\bar{q}}_{c}\cdot {\left[0,{\hat{\theta }}_{r},0,0\right]}^{T}\right)\cdot {{\bar{q}}_{c}}^{\prime }\right]}_{1‥3}$$ (21)

Where **x**_1‥3_ indicates the 3 × 1 vector obtained by ommitting the first element of a 4 × 1 vector **x**. Finally, we return the sample consisting of the translation $\bar{t}$ and the normalized rotation $\bar{q}$ between $\bar{t}$ and ${\hat{\theta }}_{c}$ :

$$\Psi {\left(\hat{I},\hat{\theta }\right)}_{\text{rotation}}=\langle \frac{\bar{q}}{\left\|\bar{q}\right\|},\bar{t}\rangle \;\bar{q}=\left\langle {\hat{\theta }}_{r}\|a\|+ba^{T},b^{T}\times a\right\rangle \;a={\hat{\theta }}_{c}-\bar{t}\;b=\left[{\hat{\theta }}_{r},0,0\right]$$ (22)

### 5.6 Best-Fit Model Selection

The linear and rotational tool transform models represent two different relationships between the translational and rotational components of corrections. We now define a metric for selecting between these two models based on how well they fit the correction data:

$$\Psi {\left(C\right)}_{\text{best}-\text{fit}}=\left\{\begin{array}{cc} \Psi {\left({\hat{I}}_{l},{\hat{\theta }}_{l}\right)}_{linear} & \text{if }\Delta_{linear}<\Delta_{rotation}\\ \Psi {\left({\hat{I}}_{r},{\hat{\theta }}_{r}\right)}_{\text{rotation}} & \text{otherwise} \end{array}\right.$$ (23)

Where ${\hat{I}}_{l},{\hat{\theta }}_{l},{\hat{I}}_{r},{\hat{\theta }}_{r}$ represent the optimal inlier points and parameter values from the linear and rotational models, respectively. The fit of the linear model is calculated as its range of values **z** projected in the model’s 1D space:

$$\Delta_{linear}=\text{range}\left(z\right)\;z=\left\{{\left(p_{t}-{\hat{\theta }}_{\mu }\right)}^{T}{\hat{\theta }}_{u}|\forall p\in \hat{I}\right\}$$ (24)

The fit of the rotational model is calculated as the range of unit vectors in the direction of each inlier point as measured from the center-of-rotation:

$$\Delta_{rotation}=1-\frac{1}{\left|\hat{I}\right|}{\left\|\underset{p\in \hat{I}}{\sum }{\left[\left(r_{p}\cdot {\left[0,1,0,0\right]}^{T}\right)\cdot {r_{p}}^{\prime }\right]}_{1‥3}\right\|}_{2}$$ (25)

where **r**_**p**_ is defined in Equation 19.

### 5.7 Evaluation

**FIGURE 9:**
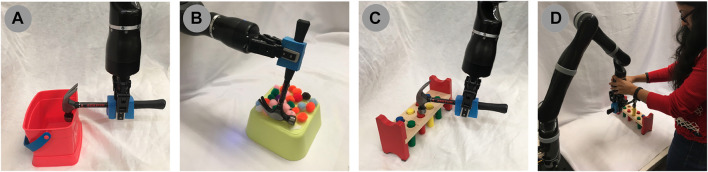
**(A)** hooking task, **(B)** sweeping task, **(C)** hammering task, and **(D)** the experimental setting.

We evaluated the transfer by correction algorithm results on a 7-DOF Jaco2 arm equipped with a two-fingered Robotiq 85 gripper and mounted vertically on a table-top surface (Figure 9D). Each evaluation configuration consisted of one task that was 1) demonstrated using the original, *“source”* tool, and 2) corrected to accommodate a novel, *replacement* tool. We describe data collection for each of these steps in the following sections.

### 5.8 Demonstrations

**FIGURE 10:**
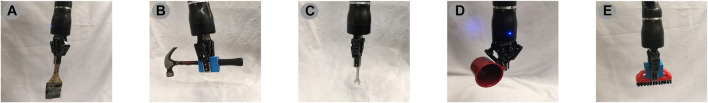
Tools **(A–C)** were used to demonstrate the three tasks shown in Figure 9, later transferred to use tools **(D,E)**. These tools exhibit a wide range of grasps, orientations, dimensions, and tooltip surfaces.

Three tasks (Figure 9) were demonstrated using three prototypical, “source” tools (Figures 10A–C), resulting in a total of nine demonstrations. Demonstrations began with the arm positioned in an initial configuration, and with the gripper already grasping the tool. Each tool’s grasp remained consistent across all three tasks. Objects on the robot’s workspace were reset to the same initial position before every demonstration. We provided demonstrations by indicating keyframes (Akgun et al., 2012) along the trajectory, each of which was reached by moving the robot’s arm to the intermediate pose. At each keyframe, the 7D end effector pose was recorded; note that this is the pose of the joint holding the tool, and *not* the pose of the tool-tip itself (since the tool-tip is unknown to the robot). We provided one keyframe demonstration for each combination of tasks and source tools in this manner, each demonstration consisting of 7–12 keyframes (depending on the source tool used) for the sweeping task, 10–11 keyframes (depending on the source tool used) for the hooking task, and 7 keyframes for the hammering task.

We represented each demonstration using a Dynamic Movement Primitive (DMP) (Schaal, 2006; Pastor et al., 2009). A DMP is trained over a demonstration by perturbing a linear spring-damper system according to the velocity and acceleration of the robot’s end-effector at each time step. By integrating over the DMP, a trajectory can then be generated that begins at the end-effector’s initial position and ends at a specified end point location. Thus, after training a DMP, the only parameter required to execute the skill is the desired end point location. By parameterizing the end point location of each DMP skill model according to object locations, the overall task can be generalized to accommodate new object configurations. We re-recorded the demonstration if the trained DMP failed to repeat the demonstration task with the source tool.

### 5.9 Corrections

Following training, the arm was reset to its initial configuration, with the gripper already grasping a new tool (Figures 10D,E). Note that these replacement objects have several surfaces that could be utilized as a tooltip (depending on the task). For example, any point along the rim of the mug (Figure 10D) would serve as the prototypical tooltip during a scooping or pouring task. In the context of the hooking and hammering tasks used in our evaluation, however, the bottom of the mug serves as a tooltip. Alternatively, the side of the mug provides a broad surface to perform the sweeping task. This range of potential tooltips on a single object highlights the benefit of using corrections to learn task-specific tooltips, rather than assume that a prototypical tooltip is appropriate for all tasks.

Objects on the robot’s workspace were reset to the same initial position as in the demonstrations; this allowed us to ensure that any corrections were made as a result of the change in tool, rather than changes in object positions. The learned model was then used to plan a trajectory in task-space, which was then converted into a joint-space trajectory using TracIK (Beeson and Ames, 2015) and executed, pausing at intervals defined by the keyframe timing used in the original demonstration. When execution was paused, it remained paused until the arm pose was confirmed. If no correction was necessary, the pose was confirmed immediately; otherwise, the arm pose was first corrected by moving the arm to the correct position. Note that this form of corrections assumes that each keyframe constitutes a statically stable state. For tasks involving unstable states, another form of interaction may be used to provide post-hoc corrections, such as critiques (Cui and Niekum, 2018).

Two poses were recorded for each correction: 1) the original end-effector pose the arm attempted to reach (regardless of whether the goal pose was reachable with the new tool), and 2) the end-effector pose following confirmation (regardless of whether a correction was given). Trajectory execution then resumed from the arm’s current pose, following the original task-space trajectory so that pose corrections were not propagated to the rest of the trajectory. This process continued until all keyframes were corrected and executed, resulting in the correction matrix **C** (Equation 2).

### 5.10 Measures

For each transfer execution, we measured performance according to a metric specific to the task:

• *Sweeping:* The number of pom-poms swept off the surface of the yellow box.
• *Hooking:* The final distance between the box’s target position and the closest edge of the box (measured in centimeters).
• *Hammering:* A binary metric of whether the peg was pressed any lower from its initial position.

### 5.11 Results

We highlight two categories of results: *Within*-task and *across*-task performance.

#### 5.11.1 Within-Task Transfer

*Within-task* performance measures the algorithm’s ability to model the corrections and perform the corrected task successfully. Transfer was performed using the transform model learned from corrections on *that same tool-task pairing*. For example, for the sweeping task model learned using the hammer, corrections were provided on the replacement tool (e.g., a mug) and then used to perform the sweeping task using that same mug. For each source tool, we evaluated performance on all three tasks using each of the two replacement objects, resulting in 18 sets of corrections (one for each combination of task, source tool, and replacement tool) per tool transform model (linear and rotational).

**FIGURE 11:**
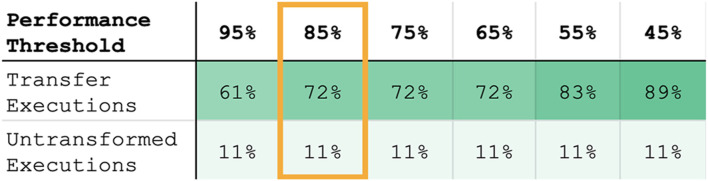
Percentage of within-task transfer executions (selected by best-fit model) and untransformed trajectories achieving various performance thresholds (defined as the % of maximum performance metric for that task, described in Section 5.10). Our proposed models result in a higher percentage of transfer executions that complete the task to a high performance threshold (e.g., sweeping ≥ 85*%* of the objects off the table). Furthermore, while the untransformed baseline produces all-or-nothing performance behavior, our models degrade gracefully, resulting in partial task completion (represented by lower % performance thresholds) even when the learned transform is non-optimal.

Using the better-performing model resulted in ≥ 85% of maximum task performance in 83% of cases. The better-performing model was selected using the best-fit metric in 72% of cases. Figure 11 lists the percentage of transfer executions (using the best-fit model) that achieve multiple performance thresholds, where best-fit results were recorded as the performance of the model returned by Equation 23.

**FIGURE 12:**
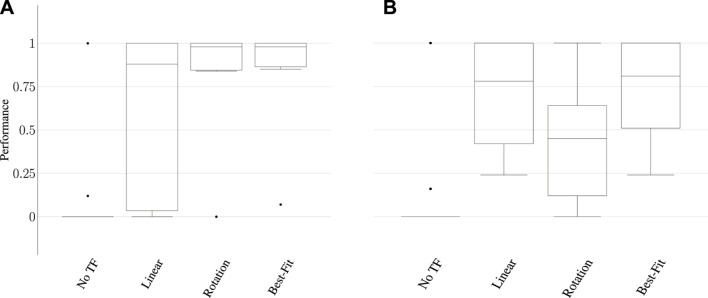
Aggregate performance results for within-task transfer using the scrub-brush **(A)** and mug **(B)** as the replacement tool. Performance was measured for each task according to the metrics in Section 5.10, and are scaled between 0–1. These results highlight the need for multiple tool transform models; while both models greatly outperform the baseline task performance (when no transform is used), note that neither model results in the *best* performance over all tasks and replacement tools. Using the best-fit metric to select the more appropriate model for each tool-task pairing resulted in the best overall performance.

**FIGURE 13:**
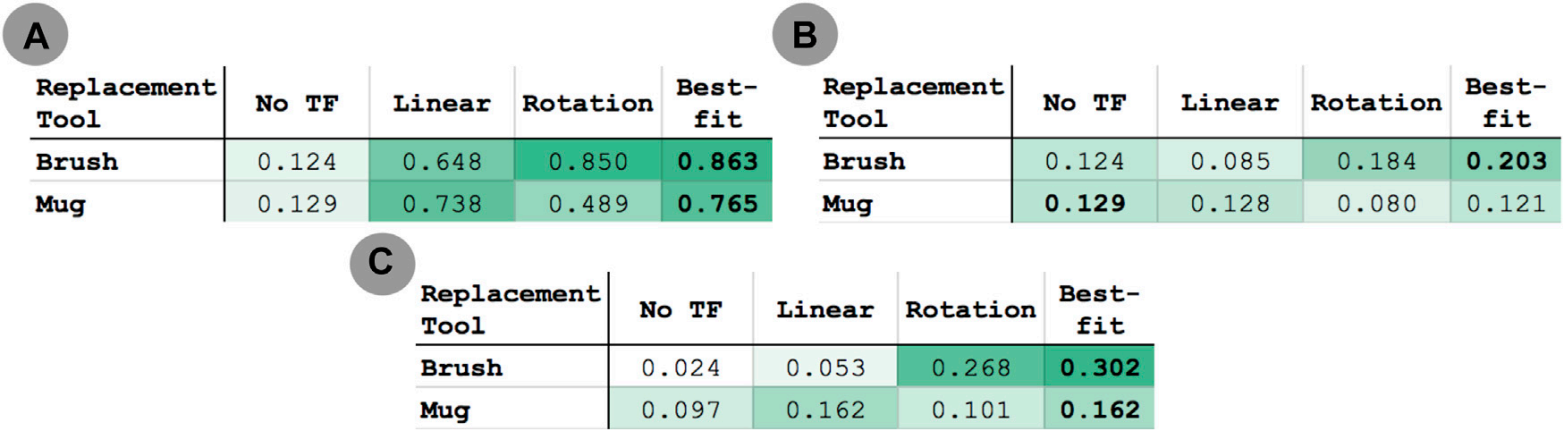
Mean performance of **(A)** within-task and **(B)** across-task transfer to the brush and mug replacement tools over all 18 transfer executions for each tool. **(C)** Mean performance of across-task transfer to the brush and mug replacement tools over the subset of transfer executions in which the transformation between source and correction tasks is similar for the source and replacement tool (10 executions for the brush, 12 for the mug). Darker cells indicate higher average performance.

We scaled the result of each transfer execution between 0 and 1, with 0 representing the initial state of the task and 1 representing maximum performance according to the metrics in Section 5.10. Figure 12 reports the performance distribution aggregated over all tasks, transferred from each of the three source tools to either the scrub-brush (Figure 10E, results in Figure 12A) or mug (pictured in Figure 10D, results in Figure 12B) as the replacement tool. The mean performance results are reported in Figure 13A, with darker cells indicating better performance. Overall, the transform returned using the best-fit metric resulted in average performance of 6.9x and 5.9x that of the untransformed trajectory when using the scrub-brush and mug, respectively, as replacement tools.

#### 5.11.2 Across-Task Transfer

*Across-task* transfer performance measures the generalizability of corrections learned on one task when applied to a *different* task using the same tool, without having received any corrections on that tool-task pairing. For example, the hooking task was learned using the hammer, and transferred to the mug using corrections obtained on the sweeping task. We evaluated 36 total transfer executions (one per combination of demonstration task, source tool, correction task (distinct from the demonstration task), and replacement tool) per tool transform model (linear and rotational).

**FIGURE 14:**
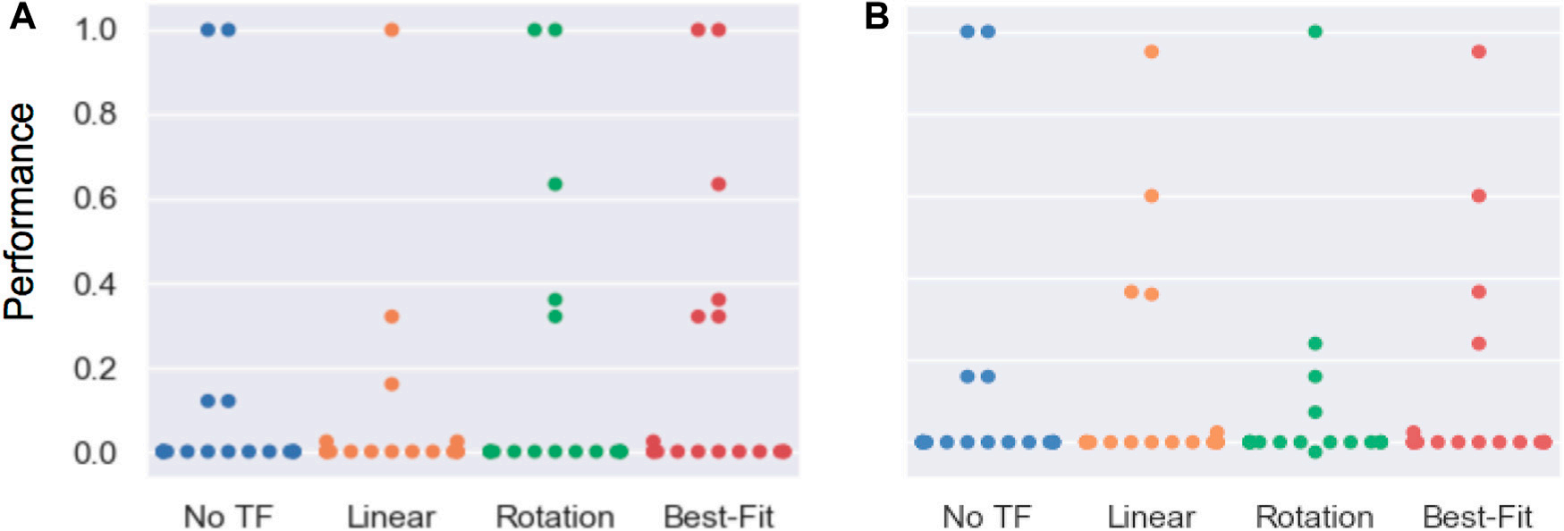
Results for across-task transfer using the scrub-brush **(A)** and mug **(B)** as the replacement tool. Performance was measured according to the metrics in Section 5.10, scaled between 0–1. These results represent the generalizability of a transform model learned on one task and then applied to a different task using the same tool. Each point represents the performance of a single transfer execution.

Figure 14 reports the performance distribution aggregated over all tasks, transferred from each of the three source tools to either the scrub-brush (Figure 14A) or mug (Figure 13B) as the replacement tool. The mean performance results are reported in Figure 13B, with darker cells indicating better performance. Overall, the transform returned using the best-fit metric resulted in average performance of 1.6*x* and 0.94*x* that of the untransformed trajectory when using the scrub-brush and mug, respectively, as replacement tools. The performance distribution is improved when using the transform learned from corrections, resulting in 2.25*x* as many task executions achieving ≥ 25% of maximum task performance.

In order to understand the conditions under which a transform can be reused successfully in the context of another task, we also report the mean performance results for a subset of the across-task executions (Figure 13C). This subset consists of only the task executions where the relative orientation is the same between 1) the source tool’s tooltips used for the source and target tasks and 2) the replacement tool’s tooltips used for the same two tasks. This subset consisted of 10 executions for the scrub-brush, and 12 for the mug. Overall, for this subset of executions, the transform returned using the best-fit metric resulted in average performance of 12.6*x* and 1.7*x* that of the untransformed trajectory when using the scrub-brush and mug, respectively, as replacement tools.

### 5.12 Discussion

**FIGURE 15:**
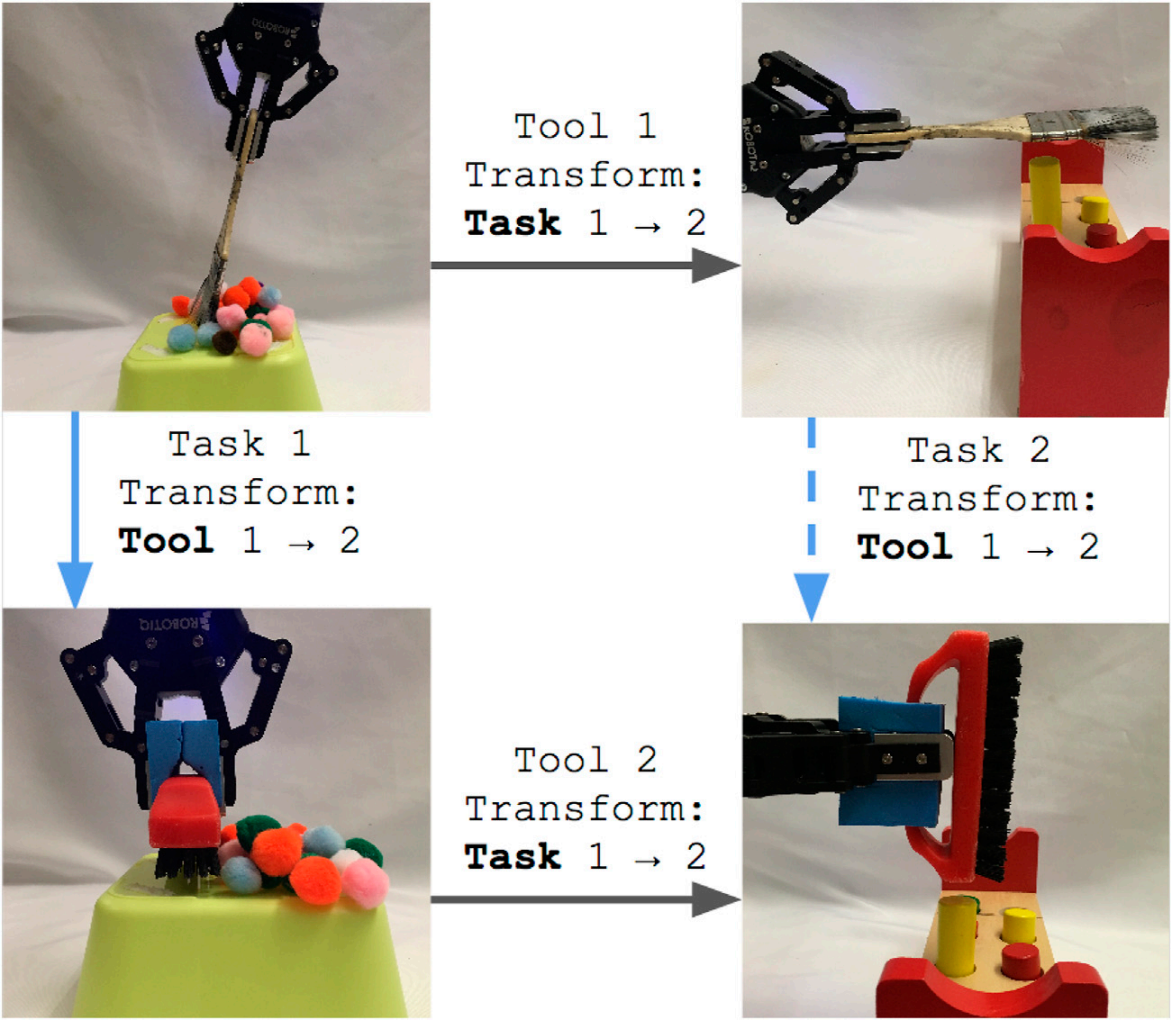
Corrections indicate the transform from tool 1 to tool 2 for the same task (indicated by the solid blue arrow). Our within-task transfer evaluation tested whether we can use corrections to sufficiently model this relationship. Different tasks may use different tooltips from the same tool (such as the different tooltips used to complete tasks 1 and 2). Our across-task evaluation tests whether the transform learned from corrections (solid blue arrow) can be reused as the transform between the two tools for another task (indicated by the dashed blue arrow).

Our within-task transfer evaluation tested whether we can model the transform between two tools in the context of the same task (represented by the solid blue arrow in Figure 15) using corrections. Our results indicate that one round of corrections typically is sufficient to indicate this relationship between tools; collectively, the linear and rotational models achieved ≥ 85% of maximum task performance in 83% of cases. Individually, the models selected by the best-fit metric achieved this performance threshold in 72% of cases. This indicates that, in general, the fit of the model itself can be used to indicate the relationship between end-effector position and orientation for a given tool/task combination.

Aside from analyzing high task performance, we are also interested in whether our approach enables graceful degradation; even if the robot is unable to complete the task fully with a new tool, ideally it will still have learned a transform that enables *partial* completion of the task. The results shown in Figure 11 demonstrate that Transfer by Correction offers robust behavior such that even when it results in sub-optimal performance, it still meets lower performance thresholds in nearly 90% of cases. In contrast, the untransformed baseline does not meet lower performance thresholds, and thus produces all-or-nothing results that lack robustness.

The primary benefit of modeling corrections (as opposed to re-learning the task for the new tool) is two-fold: First, the robot learns a transformation that reflects *how* the task has changed in response to the new tool, which is potentially generalizable to other tasks (as we discuss next). We hypothesize that in future work, this learned transform could be parameterized by features of the tool (after corrections on multiple tools). Second, since we do not change the underlying task model, but instead apply the learned transform to the resulting trajectory, the underlying task model is left unchanged. We expect that this efficiency benefit would be most evident when transferring a more complex task model trained over many demonstrations; rather than require more demonstrations with the new tool in order to re-train the task model, the transform would be applied to the result of the already-trained model.

We have also explored how well this transform generalizes to other tasks. Different tooltips on the same tool may be used to achieve different tasks, such as how the end and base of the paintbrush are used to perform sweeping and hammering tasks, respectively, in Figure 15. While we do not explicitly model the relationship between tooltips on the same tool (represented by the top grey arrow in Figure 15), they are inherent to the learned task models. A similar relationship exists for the replacement tool (represented by the bottom grey arrow in Figure 15). Our across-task evaluation seeks to answer whether the relationship between tools in the context of the first task (solid blue arrow) can be reused for a second task (represented by the dashed blue arrow) without having received any corrections on that tool/task combination (tool 2 and task 2). While we see lower performance in across-task evaluations compared to the within-task evaluations, it does improve transfer in 27.8% of across-task transfer executions (in comparison to the untransformed trajectory).

In the general case, our results also indicate that we cannot necessarily reuse the learned transformation on additional tasks, as average performance in across-task transfer is slightly worse than that of the untransformed trajectory when the mug is used as a replacement tool. This presents the question: Given a transform between two tools in the context of one task, under what conditions can that transform be reused in the context of another task *without additional corrections or training*? We do see that across-task performance is best when considering only the subset of cases where the relationship between the tooltips used in either task is similar for the source and replacement tools (in our evaluation, this is 10 of 18 executions using the brush, and 12 of 18 executions using the mug). Within this subset, across-task transfer improves performance in 41% of transfer executions. From this we draw two conclusions: 1) the transform applied to a tool is contextually dependent on the source task, target task, and tooltips of the source and replacement tool, and 2) a transform can be reused when the relationship between tooltips used in either task is similar for the source and replacement tools.

Overall, our evaluation resulted in the following key findings:

*Insight #1:* Corrections provide a sample of the *constrained* transform between the tooltip and the robot’s end-effector. This underlying constraint is task-dependent; our best-fit model results indicate that **multiple constraint types should be modeled and evaluated for each task**, with the best-fitting model used to produce the final transform output.

*Insight #2:* While the tooltip transform is task-specific, it can be applied to additional tasks under certain conditions. This is dependent on a second transform: the transform between *multiple tooltips* on the same tool. **A tooltip transform can be reused for an additional task when the transform between the tooltips used to complete 1) the corrected task and 2) the additional task are similar for the two tools.**

## 6 Conclusion

Tool use is a hallmark of human cognition and tool improvisation is a characteristic of human creativity. As robots enter human society, we expect human-like tool improvisation from robots as well. This paper makes three contributions to robot creativity in using novel tools to accomplish everyday tasks. First, it presents a high-level decomposition of the task of tool improvisation into a process of tool exploration, tool evaluation, and adaptation of task models to the novel tool. Second, it demonstrates the importance of *tooltip constraints* in guiding successful tool use throughout this process. Third, it describes a method of learning by correction: repeating a known task with an unknown tool in order to record a human teacher’s corrections of the robot’s motion.

We focused on how the relationship between the robot’s gripper and the tooltip dictates how the robot’s action model should be adapted to the new tool. A challenge in identifying this relationship is that 1) there are many candidate tooltips on each tool, and 2) for each tooltip, there exists a one-to-many relationship between the tooltip and end-effector poses that fulfill the tooltip constraint.

In this paper, we validated this one-to-many mapping through a simulated experiment in which we demonstrate a relationship between pose variations and task performance. Our experimental results indicate that the sensitivity of tooltip constraints depends on the surface of the tool being used, and that as the tool pose deviates from these constraints, the resulting effect on task performance is nonlinear.

We then examined the opposite mapping: A many-to-one mapping between pose feedback provided by a human teacher, and the optimal, underlying tooltip constraint. We developed the Learning by Correction algorithm, and demonstrated that a human teacher can indicate the tooltip constraints for a specific tool-task pairing by correcting the robot’s motion when using the new tool. We modeled the underlying tooltip constraint in two ways, using a linear and rotation model, and also present a metric for choosing the better-fitting model for a set of corrections. We demonstrated how this model of the tooltip constraint can then be used to successfully plan and execute the task using that tool with high task performance in 83% of task execusions. We also explored how this tooltip constraint model can be generalized to additional tasks using the same novel tool, without requiring any additional training data.

Overall, we expect that a focus on identifying novel tools, evaluating novel tools, and adapting task models to novel tools in accordance to tooltip constraints is essential for enabling creative tool use. Our results indicate that successful task adaptation for a new tool is dependent on the tool’s usage within that task, and that the transform model learned from interactive corrections can be generalized to other tasks providing a similar context for the new tool. Put together, these results provide a process account of robot creativity in tool use (tool identification, evaluation and adaptation), a content account (highlighting the importance of tooltips), as well as an algorithmic account of learning by correction.

### 6.1 Open Questions

In this paper, we have presented a corrections-based approach to sampling and modeling the transform resulting from a tool replacement. In doing so, we model a single, *static* transform for a particular tool/task pairing. We have evaluated how well this model transfers to other tasks using the same tool replacement. An extension of this work would consider transfer across *tools*.

We envision that a robot could not only model the transform samples obtained by interactive corrections, but also learn to generalize that model to other, similar tools. For example, after receiving corrections for one ladle for a scooping task, the robot would ideally be able to model those corrections such that it would apply to ladles of different shapes or proportions as well. We anticipate that a robot could learn an underlying relationship between visual object features (such as dimensions or concavity) and the resulting transform for that tool.

Meta-learning has been successfully applied to learning problems in computer vision domains and fully-simulated reinforcement learning problems (Duan et al., 2017; Chelsea et al., 2017). When applied to the domain of tool transfer, meta-learning would ideally enable a robot to use extensive background training to learn the common relationships between visual features and tooltips that are shared by tools within their respective categories (e.g., cups, knives, scoops). When presented with a novel category of tools, the robot would then only need demonstrations using a small number of tools within the new category in order to learn the relationship between visual features and tooltips within that category. However, as demonstrated in this paper, tooltips are task-specific; within a single tool, the tooltip used to complete one task (e.g., the surface of a hammer used to hammer a nail) is not necessarily the same as the tooltip used to complete another task (e.g., the side of the hammer may be used to sweep objects off a surface, or the claw-end of the hammer may be used to remove a nail). This lack of task-specific training data presents a challenge for future work, as relying on a dataset containing a single, canonical tooltip for each tool would fail to capture the task-contextual nature of tool use.

Finally, this paper has explored one method of interaction to enable a human teacher to provide corrections to the robot. However, in human-in-the-loop learning problems, the ideal interaction type is dependent on the teacher’s role in the learning system, and the context in which the robot is used (Cui et al., 2021). For example, the teacher may not have time to correct every step of the robot’s action, or may instead prefer to provide corrections only after the robot has tried and failed to complete a task. We anticipate that future work may enable a robot to obtain correction data from a broader set of interaction types.

## Data Availability

The raw data supporting the conclusion of this article will be made available by the authors, without undue reservation.
